# Less is More in Antithrombotic Therapy for Durable Left Ventricular Assist Devices

**DOI:** 10.2174/011573403X307310240404062647

**Published:** 2024-04-15

**Authors:** Matthew T. Brown, Nanette K. Wenger

**Affiliations:** 1 Department of Medicine, Division of Cardiology, Emory University School of Medicine, Atlanta, Georgia

**Keywords:** Aspirin, atherosclerotic cardiovascular disease, vitamin K antagonist, left ventricular assist device, HeartMate 3, mechanical heart valves

## 1 INTRODUCTION

Since the publication of our manuscript, there has been an important update regarding the use of aspirin among durable Left Ventricular Assist Devices (LVAD) [1]. To date, the antithrombotic regimen for LVADs has included both aspirin and a vitamin K antagonist, similar to the recently addended recommendations for mechanical heart valves [2, 3]. This pharmacologic regimen has remained consistent, as have major bleeding episodes, despite advances in technology to a fully magnetically levitated frictionless pump system featured in the HeartMate3 (HM3) LVAD shown to significantly reduce instances of pump thrombosis and disabling stroke [4-6].

## 2 ANTITHROMBOTIC THERAPY IN HM3 LVADS

In light of the changing role of aspirin and efforts to minimize bleeding, a recent investigation entitled Antiplatelet Removal and Hemocompatibility Events with the HeartMate3 Pump, known as the ARIES-HM3 trial, randomized 589 patients with HeartMate3 (HM3) LVADs to receive vitamin K antagonists and either aspirin (100 mg/d) or placebo in a 1:1 double-blind fashion [7]. The composite primary endpoint was assessed for noninferiority of placebo and focused on survival free of hemocompatibility-related adverse events, including stroke, pump thrombosis, major bleeding, or arterial peripheral thromboembolism at 12 months. Secondary endpoints included nonsurgical bleeding events, thrombotic events, and rates of hospitalization due to nonsurgical bleeding, which allowed for estimates of number of hospital days averted and cost of bleeding episodes.

**Fig. (1):**
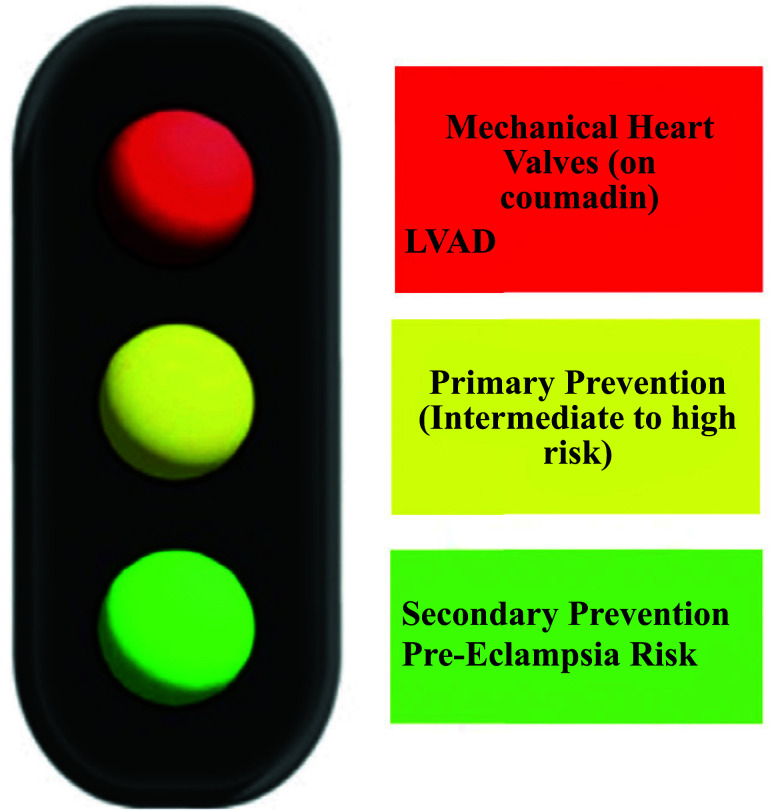
Incorporating aspirin avoidance in LVAD therapy into the schemata for aspirin’s roles in cardiovascular disease.

More patients reached 12-month survival free of stroke, pump thrombosis, major bleeding, or arterial peripheral thromboembolism in the placebo group than in the aspirin group [68.1% (186/273) *vs*. 74.2% (201/271)], largely driven by a reduction in bleeding events in the placebo group (22.5% *vs*. 28.2%). This absolute between-group difference of 6.0% in hemocompatibility-related adverse events met the threshold for noninferiority of the placebo. This noninferiority held true across all subgroups, including the history of coronary stenting, coronary artery bypass grafting, diabetes, atrial fibrillation, and ischemic etiology of heart failure. There were fewer first nonsurgical bleeding events in the placebo than aspirin group at 24 months (30.0 *vs*. 42.4, *p* = 0.1) and this significant reduction held true for cumulative (first and recurrent) moderate and severe events, including gastrointestinal sources with a Relative Risk (RR) of 0.66 (95% CI, 0.51 – 0.85; *p* = 0.002). Rates of nonsurgical bleeding hospitalizations were lower in the placebo group compared to the aspirin group (13.6 *vs*. 23.9 events per 100 patient-years, *p* = 0.002), which correlated with a cost reduction of 41%, approximately $380,000, and 293 (47%) fewer days spent in the hospital for bleeding. With no instances of pump thrombosis or arterial peripheral thromboembolism within 12 months, thrombotic events solely evaluated rates of ischemic stroke, which were similar between placebo and aspirin groups [1.6% *vs*. 2.7%, RR 0.58 (95% CI, 0.21 – 1.58); *p* = 0.29] as were rates of ischemic and hemorrhagic strokes combined [1.9% *vs*. 3.7%, RR 0.52 (95% CI, 0.21 – 1.30); *p* = 0.16] (Fig. **1**).

## CONCLUSION

While the study had a limited number of females (23%), largely recruited from North America (85%), excluded patients with early surgical complications, and focused solely on the HeartMate3 LVAD, it had provided convincing evidence for aspirin avoidance in an antithrombotic regimen containing vitamin K antagonists even across a variety of high-risk subgroups. Similar to recent data lending to aspirin’s tempered role in atherosclerotic cardiovascular disease management, the ARIES-HM3 trial demonstrated a significant reduction in nonsurgical bleeding endpoints without any difference in thrombotic events [8-13]. These data provide evidence for yet another redefinition of the role of aspirin in cardiovascular disease and are important to consider in the context of our original review.

## AUTHORS’ CONTRIBUTIONS

It is hereby acknowledged that all authors have accepted responsibility for the manuscript's content and consented to its submission. They have meticulously reviewed all results and unanimously approved the final version of the manuscript.

## LIST OF ABBREVIATIONS

ARIES-HM3: Antiplatelet Removal and Hemocompatibility Events with the HeartMate3 Pump
HM3: HeartMate 3
LVAD: Left Ventricular Assist Device

## References

[1] Brown M.T., Bortfeld K.S., Sperling L.S., Wenger N.K. (2023). Redefining the roles of aspirin across the spectrum of cardiovascular disease prevention.. Curr. Cardiol. Rev..

[2] Consolo F., Lucchetti R.M., Tramontin C., Lapenna E., Pappalardo F. (2019). Do we need aspirin in HeartMate 3 patients?. Eur. J. Heart Fail..

[3] Otto C.M., Nishimura R.A., Bonow R.O. (2021). 2020 ACC/AHA guideline for the management of patients with valvular heart disease: A report of the American College of Cardiology/American Heart Association Joint Committee on clinical practice guidelines.. Circulation.

[4] Shah P., Tantry U.S., Bliden K.P., Gurbel P.A. (2017). Bleeding and thrombosis associated with ventricular assist device therapy.. J. Heart Lung Transplant..

[5] Del Rio-Pertuz G., Nair N. (2023). Gastrointestinal bleeding in patients with continuous‐flow left ventricular assist devices: A comprehensive review.. Artif. Organs.

[6] Tedford R.J., Leacche M., Lorts A., Drakos S.G., Pagani F.D., Cowger J. (2023). Durable mechanical circulatory support.. J. Am. Coll. Cardiol..

[7] Mehra M.R., Netuka I., Uriel N. (2023). Aspirin and hemocompatibility events with a left ventricular assist device in advanced heart failure.. JAMA.

[8] Agarwal N., Mahmoud A.N., Patel N.K. (2018). Meta-analysis of aspirin *versus* dual antiplatelet therapy following coronary artery bypass grafting.. Am. J. Cardiol..

[9] Arnett D.K., Blumenthal R.S., Albert M.A. (2019). 2019 ACC/AHA guideline on the primary prevention of cardiovascular disease: Executive summary: A report of the American College of Cardiology/American Heart Association task force on clinical practice guidelines.. Circulation.

[10] Davidson K.W., Barry M.J., Mangione C.M. (2022). Aspirin use to prevent cardiovascular disease.. JAMA.

[11] Gragnano F., Cao D., Pirondini L. (2023). P2Y12 inhibitor or aspirin monotherapy for secondary prevention of coronary events.. J. Am. Coll. Cardiol..

[12] Knijnik L., Fernandes M., Rivera M. (2021). Meta-analysis of duration of dual antiplatelet therapy in acute coronary syndrome treated with coronary stenting.. Am. J. Cardiol..

[13] O’Donoghue M.L., Murphy S.A., Sabatine M.S. (2020). The safety and efficacy of aspirin discontinuation on a background of a P2Y 12 inhibitor in patients after percutaneous coronary intervention.. Circulation.

